# Incidence and Virulence Factor Profiling of *Vibrio* Species: A Study on Hospital and Community Wastewater Effluents

**DOI:** 10.3390/microorganisms11102449

**Published:** 2023-09-29

**Authors:** Mashudu Mavhungu, Tennison O. Digban, Uchechukwu U. Nwodo

**Affiliations:** 1 Patho-Biocatalysis Group, Department of Biochemistry and Microbiology, University of Fort Hare, Private Bag X1314, Alice 5700, South Africadigbant@gmail.com (T.O.D.); 2 Department of Biochemistry and Microbiology, University of Fort Hare, Alice 5700, South Africa

**Keywords:** virulence genes, hospital effluent, *Vibrio* species, cholera, TCBS, South Africa

## Abstract

This study aimed to determine the incidence and virulence factor profiling of *Vibrio* species from hospital wastewater (HWW) and community wastewater effluents. Wastewater samples from selected sites were collected, processed, and analysed presumptively by the culture dependent methods and molecular techniques. A total of 270 isolates were confirmed as *Vibrio* genus delineating into *V. cholerae* (27%), *V. parahaemolyticus* (9.1%), *V. vulnificus* (4.1%), and *V. fluvialis* (3%). The remainder (>50%) may account for other *Vibrio* species not identified in the study. The four *Vibrio* species were isolated from secondary hospital wastewater effluent (SHWE), while *V. cholerae* was the sole specie isolated from Limbede community wastewater effluent (LCWE) and none of the four *Vibrio* species was recovered from tertiary hospital wastewater effluent (THWE). However, several virulence genes were identified among *V. cholerae* isolates from SHWE: *ToxR* (88%), *hylA* (81%), *tcpA* (64%), *VPI* (58%), *ctx* (44%), and *ompU* (34%). Virulence genes factors among *V. cholerae* isolates from LCWE were: *ToxR* (78%), *ctx* (67%), *tcpA* (44%), and *hylA* (44%). Two different genes (*vfh* and *hupO*) were identified in all confirmed *V. fluvialis* isolates. Among *V. vulnificus*, *vcgA* (50%) and *vcgB* (67%) were detected. In *V. parahaemolyticus*, *tdh* (56%) and *tlh* (100%) were also identified. This finding reveals that the studied aquatic niches pose serious potential health risk with *Vibrio* species harbouring virulence signatures. The distribution of virulence genes is valuable for ecological site quality, as well as epidemiological marker in the control and management of diseases caused by *Vibrio* species. Regular monitoring of HWW and communal wastewater effluent would allow relevant establishments to forecast, detect, and mitigate any public health threats in advance.

## 1. Introduction

The unceasing emergence and re-emergence of bacteria to pathogenic status remains a threat to public health with concomitant adverse effects on the economic prosperity of any society [1,2]. Threats to human health and the change in the ecosystems have surged as a result of climate change [3]. The downstream results of these changes affect broader environmental systems, which have an impact that is either actively or passively linked to health. [4]. The consequence of the change is transforming disease forms for some illnesses that are primarily subject to environmental fluctuations, and a pertinent class of anthropological pathogens currently undergoing rapid growth is *Vibrio* [5]. As a result of climate change, precipitation patterns and the temperature of sea surfaces are varied with the rising incidence of *Vibrio*-linked diseases in aquatic environments affecting both humans and aquatic animals [6]. Vibriosis (infection vibrios) in humans has been documented as cholera or non-cholera infection [7].

According to the World Health Organization (WHO), approximately 4 million cases of cholera are reported each year from endemic countries with more than 100,000 fatalities [8]. However, global *Vibrio* infection surveillance is limited, principally for developing countries, stemming from under-reporting or failure to report infections, variations in reporting methods, and absence of an international epidemiology structure [9]. Accessibility to potable water for drinking and domestic use remains a difficulty in rising nations such as South Africa, forcing some rural communities to utilize microbiologically polluted river water for personal and household functions, thereby posing a public health concern [10].

*Vibrio* species are inclusive in the category of emerging and re-emerging bacterial pathogen, while *V. cholerae*, *V. parahaemolyticus*, *V. vulnificus*, and *V. fluvialis* have been at the fore [11]. There are over a hundred species in the *Vibrio* genus that are common inhabitants of aquatic and marine environments thriving in low to moderate salinities. [12]. Of the many described *Vibrio* species, at least 12 species have been reported of having the propensity to cause infections in humans [13]. Evidently, it has been established that mobile genetic components in their genomes alter their phenotypic ability to adapt to the environment and results in pathogenicity within a host. [14,15]. Cholera remains a major public health problem, primarily in resource constrained nations where accessibility to portable drinking water and suitable sanitation cannot be rendered to all inhabitants [16]. Non-cholera causing vibrios, including *V. parahaemolyticus*, *V. alginolyticus*, *V. vulnificus*, and non-toxigenic (non-O1 or non-O139 serogroup of *V. cholerae*) cause vibriosis, giving rise to different medical cases. Clinical conditions linked to *Vibrio* infection range from minor gastroenteritis to life-threatening necrotizing fasciitis, putrid wound infections, blood disease, liver dysfunction, and acute gastroenteritis inclusive in the disease progression [17,18]. Virulence factors in *Vibrio* species required to initiate infection in susceptible host cells include siderophores desirable in foraging iron, haemolysin that lyse red blood cells including cellular membranes, capsular polysaccharide that assist in resisting opsonisation and escape complement fixation, pili and surface proteins to increase attachment, and flagella-mediated motility [19].

However, owing to *Vibrio’s* highly flexible genomic structure, the risk of horizontal gene transfer (virulence) between pathogenic and environmental *Vibrio* strains is high, increasing the number of pathogenic strains of *Vibrio* in aquatic environments [20]. The O1 and O139 serogroup of *V. cholerae* have been among the leading virulent groups and are known to harbour cholera toxin [21,22,23]. The pathogenicity of *Vibrio* depends on the combination of virulence factors such as cholera toxin (*ctxA*) and the aptitude of *V. cholerae* to colonize the colon with the colonization factor (*tcpA*) toxin co-regulated pilus along with *zot* (generally is involved in invasion) [24]. These three virulence factors (*ctxA*, *tcpA*, and *zot*) are responsible for the observed rice watery diarrhoea in humans [25]. The research on *Vibrio* continues to be on the rise and the reservoirs of these pathogens have been attributed, mostly, to the environment including municipal wastewaters and other surface waters. South Africa being a semi-arid region has a shortage of water distribution especially in the deep rural settings. There are also a number of factors that contribute to the decline of water quality in South Africa, like agricultural and industrial activities, poor functional state of treatment plants, and also impaired pipes that empty their contents into environment contaminating nearby aquatic niches [26]. Numerous occurrences of disease outbreaks like diarrhoea have been documented in various provinces of South Africa, with wastewater effluents as the key source [27]. Although hospital wastewater has lately been on the spotlights as a crucial point for the emergence of pathogens with varied characteristics [28], a paucity of information abounds on hospital wastewater effluent as a reservoir for pathogenic *Vibrio* species. Consequently, this study was premised to determine the incidence and virulence factor profiling of *Vibrio* species from both hospital and rural community wastewater within the rural Eastern Cape of South Africa.

## 2. Materials and Methods

### 2.1. Area of Study

The study was conducted in two municipalities of the rural Eastern Cape: Buffalo City and Amathole. On the province’s eastern shore, there is a metropolitan municipality called Buffalo City. The municipality instituted as a local municipality in the year 2000 after South Africa’s restructuring of municipal regions was named as a result of the Buffalo River. Auto industry is important to the region, with a well-developed manufacturing base. Amathole District Municipality is situated in the mid-region of the Eastern Cape and stretches on the Fish River Mouth through the Eastern Seaboard to the South of Hole in the Wall along with the Wild Coast. It is also abutted to the north via the Amathole Mountain Range. Due to the significant amount of wastewater produced each day, the municipal wastewater treatment facility was chosen. The hospitals selected were referrals of secondary and tertiary health institutions with a significant number of wards and other facilities for numerous patients, resulting in large amount of wastewater production. Sites and geographical coordinates of the study area are presented in Table 1.

### 2.2. Sample Collection

A total of thirty-six wastewater samples were collected weekly, spanning a period of three months (February 2018 to April 2018). Twelve (12) samples were collected from each sampling site. The samples were conveyed in cooler ice package to Patho-Biocatalysis laboratory situated in University of Fort Hare. All the wastewater samples were analysed within 2 h of collection, while the remaining samples were aliquoted into smaller collection bottles and kept at 4 °C prior to the completion of all analyses.

### 2.3. Sample Processing, Cultivation and Identification of Vibrios

A standard membrane filtration technique was utilized in processing all the samples [29]. A ten-fold serial dilution of the samples was carried out by diluting 30 mL of the samples in 270 mL of autoclaved distilled water. Using a vacuum pump, wastewater (0.1 L) was filtered through 0.45μm white gridded cellulose ester membrane filters (Merck, Johannesburg South Africa). The membrane filter paper was placed into test tubes containing alkaline peptone water (for enrichment) and incubated aerobically at 37 °C overnight. A loopful of alkaline peptone culture was sub-cultured onto thiosulphate citrate bile salts sucrose (TCBS, Himedia, South Africa) and incubated for another 24 h at 37 °C. Random selections of four to five yellowish and greenish colonies from each culture plate were purified on fresh nutrient agar plates. The purified isolates were used for the molecular analyses and the extras were stored in glycerol cryotubes at −20 °C prior to other studies.

### 2.4. Molecular Identification

#### Deoxyribonucleic Acid (DNA) Extraction

DNA extraction was carried out according to a previously described method [30], but with minor adjustment. After incubation on overnight nutrient agar (Bio-lab), pure colonies were picked and emulsified in 200μL of sterile water. Cells were then lysed by boiling at 100 °C for 15 min on a digital Accu-Block, (Lasec, Capetown, South Africa). Separation of cell debris from DNA was performed by centrifugation at 13,400× *g* for 10 min in a microcentrifuge (Lasec, Capetown, South Africa). Thereafter, the lysate was dispensed into autoclaved Eppendorf tubes and stored at −20 °C chiller to avoidt DNA degradation.

### 2.5. Vibrio isolates Confirmation

*Vibrio* isolates were validated by PCR, utilizing the 16SrRNA variable fragment as a target. All *Vibrio* isolates were further categorized as *V. cholerae*, *V. fluvialis*, *V. vulnificus*, and *V. parahaemolyticus*. The cocktail comprised 9.5 μL of Master-mix (Biolabs, New England), 0.5 μL of primers (forward and reverse) (Inqaba Biotech, Pretoria, South Africa), 2.5 μL of PCR water, and 2.5 μL of DNA template, totalling 15 μL reaction. PCR was carried out in a T1000 Touch Thermal Cycler (Bio-Rad, Johannesburg, South Africa). Amplified products were resolved by electrophoresis at 90 V for 55 min and visualized in a UV transilluminator (Alliance 4.7 Uvitec, Cambridge, UK). The targeted genes, oligonucleotide sequences, amplicon sizes, and PCR-cycling conditions of the *Vibrio* species are shown in Table 2.

### 2.6. Virulence Genes Detection

The detection of genes coding for virulence among the *Vibrio* species was carried out using PCR. Toxin-coregulated pilus (*TCP*), zonula occludens toxin (*zot*), toxin regulon (*toxR*), El Tor haemolysin (*hylA*), *Vibrio* pathogenicity island (*VP*I), cholera toxin gene (*ctx*), and outer membrane proteins (*ompU*) were the targeted virulence signatures for *V. cholerae*. For *V. vulnificus* virulence genes, *vcgA* and *vcgB* were investigated. The haeme-utilization protein gene (*hupO*), heat stable enterotoxin (*stn*), extracellular haemolysin gene (*vfh*), and *V. fluvialis* protease gene (*vfpA*) were the targeted virulence genes for *V. fluvialis*. Finally, the thermostable direct haemolysin (*tdh*), thermostable related haemolysin (*trh*), and thermolabile haemolysin gene (*tlh*) were the targeted genes in *V. parahaemolyticus*. The PCR mixture comprised of a multiplex with conditions as follows: an initial denaturation of 94 °C for 4 min and 35 cycles of denaturation at 94 °C for 45 s, annealing (52 °C and 62 °C), elongation at 72 °C for 85 s, and final extension was achieved at 72 °C for 7 min. The oligonucleotide primers, various annealing conditions, and the justification for selecting these virulence genes was subject to their previous detection in earlier studies as referenced in Table 3.

## 3. Results

**Figure 1:**
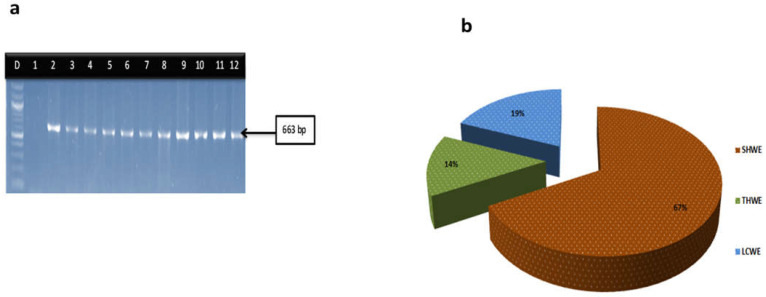
(**a**) PCR gel representatives of *Vibrio* genus. Lane D: DNA Ladder (100 bp), lane 1:. negative control, lane 2: Positive control (663bp), lane 3–12: isolates positive to 16SrRNA gene (663 bp). (**b**) Distribution of the *Vibrio* genus isolates among the three sampling sites.

During the course of the three months of sampling, 378 probable *Vibrio* isolates were recovered. Of these, 270 (71%) were confirmed *Vibrio* (genus) isolates by targeting the 16SrRNA genes. However, further analyses revealed that SHWE, LCWE, and THWE sampling sites accounted for 67%, 19%, and 14% of the *Vibrio* isolates respectively. Delineation of the *Vibrio* genus into species is given as follows: *V. cholerae* 73 (27%), *V. parahaemolyticus* 25 (9.1%), *V. vulnificus* 12 (4.1%) and *V. fluvialis* 8 (3%). The agarose gel electrophoresis of the *Vibrio* genus and the distribution of the confirmed *Vibrio* isolates are shown in Figure 1a,b respectively.

### 3.1. Prevalence of Vibrio Species

**Figure 2:**
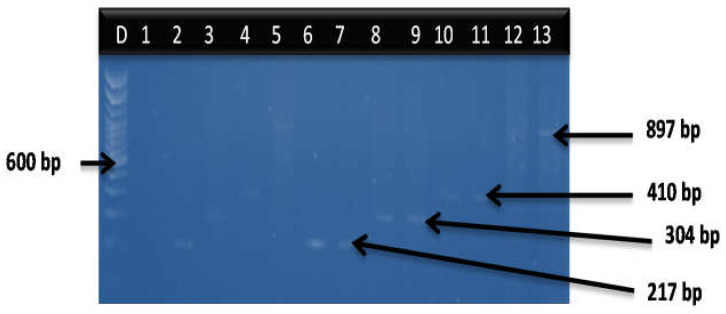
PCR multiplex gel representatives of the *Vibrio* species. Lane D: DNA ladder, lane 1: negative control. Lanes 2,6,7: positive isolates for *V. fluvialis* (217 bp). Lanes 3,8,9: positive *V. cholerae* isolates (304 bp). Lanes 4,10,11: positive *V. vulnificus* isolates (410 bp). Lanes 12, 13: positive *V. parahaemolyticus isolates* (897 bp).

Delineation of the *Vibrio* genus into species from SHWE sampling site is given as follows: *V. cholerae* (24%), *V. parahaemolyticus* (9%), *V. vulnificus* (4%) and *V. fluvialis* (3%). All four delineated *Vibrio* species were not detected in THWE, while 3% of *V. cholerae* was detected in LCWE. The gel representative of the delineated four amplified *Vibrio* species is shown in Figure 2.

### 3.2. Distribution of Virulence Genes among the Vibrio Species

**Figure 3:**
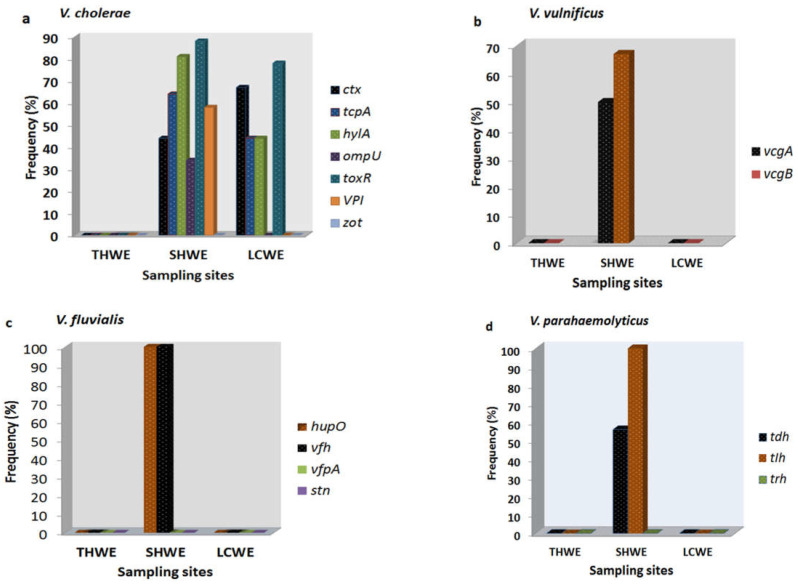
(**a**) shows the frequency of virulence genes detected among *V. cholerae* isolates in the sampling sites, (**b**) shows the frequency of the dual virulence genes among isolates of *V. vulnificus* at the three sites, (**c**) shows the frequency of virulence genes (*hupO* and *vfh*) among *V. fluvialis* isolates in the sampling sites and (**d**) shows the frequency of the *tdh* and *tlh* among *V. parahaemolyticus* isolates in the three sites.

**Figure 4:**
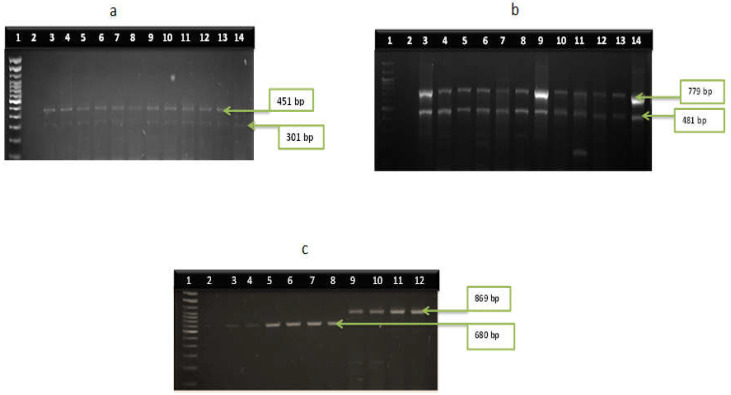
Gel multiplex representatives among *V. cholerae* isolates (**a**) lane 1: DNA molecular ladder (100 bp), lane 2: negative control; lane 3–14: positive *ctx* gene (301 bp) and *tcpA* genes (451), (**b**), lane 1: DNA molecular ladder (100 bp), lane 2: negative control; lane 3–14: positive isolates for *toxR* gene (779 bp) and *hylA* gene (481 bp), (**c**), lane 1: molecular DNA ladder (100 bp), lane 2: negative control, lane 3–8: positive isolates for *ompU* gene (869 bp), lane 9–12: positive isolates for *VPI* genes (680 bp).

**Figure 5:**
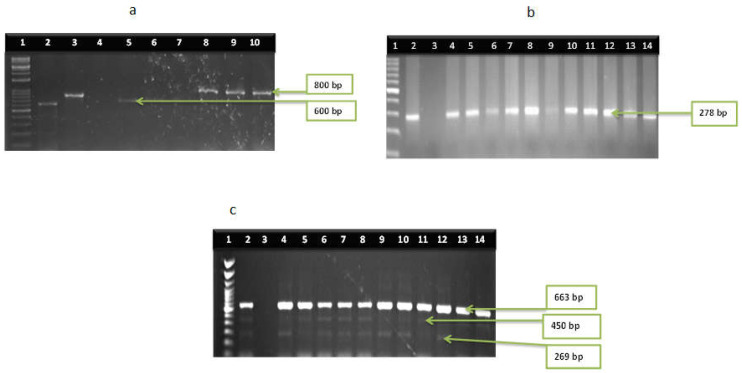
(**a**) Gel multiplex representative isolates of *V. fluvialis.* Lanes 3,8,9 and 10 showing *hupO* gene (800 bp) and lanes 2 and 5 showing *vfh* gene (600 bp). However, lanes 4, 6 and 7 are negative controls. (**b**) gel multiplex electrophoresis of *V. vulnificus* isolates showing *vcgA* gene and *vcgB* gene with 278 bp respectively, (**c**) gel multiplex electrophoresis of *V. parahaemolyticus* isolates. Lane 1: DNA ladder (100 bp), lane 3: negative control, lane 2, 4–14: positive isolates for 16srRNA gene (663 bp), *Tdh* gene (269 bp) and *TIh* gene (450 bp).

Among the confirmed *Vibrio* species, thirteen of the sixteen virulence genes were identified. *V. cholerae* harboured the following amplified genes; *ctx* (44%), *tcpA* (64%), *hylA* (81%), *ompU* (34%), *toxR* (88%), and *VPI* (58%) recovered from SHWE, while *ctx*, *tcpA*, *hylA,* and *toxR* were detected in 67%, 44%, 44%, and 78% of *V. cholerae* isolates recovered from LCWE. Two different genes (*vfh* and *hupO*) were identified in all *V. fluvialis* isolates while the *vfpA* gene was absent. Virulence genes (*tdh* and *tlh*) were detected in the proportion of 56% and 100% among *V. parahaemolyticus* from the study. Two virulence genes *vcgA* (50%) and *vcgB* (67%) were found in *V. vulnificus* isolates. The frequency of virulence genes among the isolates is presented in Figure 3a–d, while the gel representatives for the detection of the virulence genes are presented in Figure 4a–d and Figure 5a–d respectively.

## 4. Discussion

The unabated rise in the demand for water in industries, agriculture, and sustenance of urban and rural population has culminated in considerable water scarcities impacting developing countries [45]. However, an age long approach for water scarcity amelioration includes the recycling and treatment of wastewater for domestic and other uses. Wastewater contains diverse pathogens that have detrimental consequences on human health [46]. The presence of organic materials, by products from chemicals and pharmaceuticals, as well as biological infectious organisms in hospital wastewater poses a threat to both humans and animals. [47]. South Africa’s microbial pollution of water sources is mainly caused by poor management of wastewater treatment plants and uncontrolled sewage discharge. In this study, *V. vulnificus, V. cholerae, V. fluvialis,* and *V. parahaemolyticus* were isolated using a culture-dependent technique and molecular approach. This is similar to earlier reports that detected these *Vibrio* species from the aquatic environment [48,49,50,51]. However, the residual isolates of > 50% not detected in this study were anticipated to belong to other species not part of the research design. However, a previous study [52] has documented twelve known *Vibrio* species known to cause infection in humans, of which the *Vibrio* species detected in our study are inclusive and also considered most significant. The four *Vibrio* species were, however, detected in the SHWE while only *V. cholerae* isolates were recovered from LCWE. Hospital wastewater contains a pool of microbes that are considered major contributors to emerging microbial contamination. In the rural area of this study, more serious cases like surgeries and other life-threatening conditions are usually carted for in the tertiary health centres which also serve as referrals from both primary and secondary health centres. Nonetheless, the *Vibrio* species identified in this study were not detected in the THWE. The first point of call for patients in the rural communities is usually the primary or secondary health centres. It is also feasible that a lesser number of patients harbouring the organisms were admitted into the tertiary health centre during the period of study; hence the lower number of *Vibrio* isolates recovery from the THWE.

Virulence is defined as the ability of any bacteria to invade a host cell in order to initiate a disease condition. Most organisms need an assortment of virulence factors or genes to induce infection. These virulence factors can either be secretory, membrane associated, or cytolytic in nature [53]. The majority of microorganisms’ pathogenicity and infection severity are known to rise through the acquisition of virulence genes, exacerbating their impact on their susceptible host [54]. Both virulence factors and bacterial toxins contribute to pathogenicity by increasing both the infectiousness of pathogenic bacteria and antimicrobial resistance, which in turn limits the effectiveness of available treatments. The *Vibrio* species that have been linked with diseases in animals and humans harbour a collection of virulence genes.

Although, environmental isolates are typically devoid of virulence-linked genes present in the clinical isolates, studies have shown the frequency of such genes or their homologues are acquired via horizontal transfer actions, owing to the flexibility of their genomes [55,56]. In this study, *V. cholerae* was found to harbour a variety of genes. *Ctx* gene is an important virulence factor of *V. cholerae* and *is* responsible for the production of cholera toxin, accountable for diarrhoea among people with cholera [57]. The toxin regulated pilus (*tcpA*) is a type IV pilus encoded in *VPI* enabling *V. cholerae* to establish colonization in the gut and cause disease diarrhoea, while the *hylA* gene is linked with red blood cell lysis in the infected host cells [58,59]. The *ompU* gene is an important outer membrane protein that allows *V. cholerae* to adhere to the host intestinal epithelium during the course of inducing disease [60]. *ToxR* is a transcriptional activating factor that controls the expression of other essential virulence genes such as toxin-coregulated pilus and accessory colonization factor as well as the *ctx* gene [61,62]. *Vibrio* pathogenicity island (*VPI*) is a large chromosomal region responsible for virulence genes acquired through horizontal gene transfer, which functions pertinently in the production of cholera toxin and intestinal colonization in *V. cholerae* within the host intestine during the course of disease development [63]. In the absence of the cholera toxin, *zot* is one of the most important toxins in *V*. cholerae [64]. *Zot* is a conserved protein in filamentous vibriophage and has been observed as a putative toxin in *V. cholerae* [65]. In this study, *V. cholerae* harboured all aforementioned six virulence genes (*ctx*, *tcpA*, *hylA*, *ompU*, *toxR*, and *VPI*) and was recovered from SHWE, while the same genes, with the exceptions of *VPI* and *ompU*, were observed in LCWE. The presence of *V*. cholerae with virulence markers in SHWE and LCWE can be detrimental to humans when the wastewater is discharged through faulty treatment plants or leaked sewages into adjourning aquatic environments, thereby increasing the risk of contamination. Previous studies have detected *V. cholerae* isolates with virulence traits isolated from environmental and clinical sources [66,67].

*V. vulnificus* has a global dissemination and has been reported as an emerging food-borne pathogen, causing diarrheal illnesses and extraintestinal infections [68]. Also in this study, virulence correlated gene (*vcg*) was detected among the *V. vulnificus* isolates. The *vcg* gene has been reported to be useful in differentiating potentially virulent and avirulent strains [69]. The clinical isolates harbouring the *vcgA* were less detected in our study as compared to the environmental strains possessing the *vcgB* gene. This finding is comparable with a previous study that also detected more of the *vcgB* genotype denoting the less virulence strains [70]. Virulence factors that have been documented in the pathogenicity of *V. fluvialis* include haem-utilization protein (*hupO*), which is linked with virulence expression via stimulation of haemolysin production and resistance to oxidative stress [71,72]. Our study revealed the presence of *hupO* and *vfh* genes in every *V. fluvialis* isolate detected in SHWE. Additionally, *V. fluvialis* infection has increased public health risks globally and occurs in areas where people typically eat raw or undercooked seafood [73]. *Vfh* has a broad spectrum of cytocidal activity and a virulence trait harboured by *V. fluvialis,* inducing inflammatory diarrhoea in susceptible human hosts as well as eliciting the lysis of erythrocytes [74]. A similar trend was observed in a previous finding [36] of the detection *vfh* and *hupO* genes among *V. fluvialis* isolates. *V. parahaemolyticus* infection in humans has been frequently documented in coastal areas, attributed to the high consumption of sea products and frequent contact with *Vibrio* infected estuarine water bodies [75]. *V. parahaemolyticus* strains are detected by multiple virulence genes, comprising *tih* species specific virulence factors which codes for thermolabile haemolysin [76]. Furthermore, this species of *Vibrio* is also known to possess thermostable direct haemolysin (*tdh*), as well as thermolysin related haemolysin, which are important in lysing erythrocytes [77]. From our study, all *V. parahaemolyticus* isolates from SHWE sites harboured the *tlh* gene, while 56% of the isolates had thermolabile related haemolysin gene. This infers the clinical significance of aquatic *V. parahaemolyticus* pathogenicity towards humans. This finding is at variance with a previous report that detected approximately 15.6% of *tdh* gene in the aquatic environment [78].

The detection of virulence genes among *Vibrio* species in our study further reaffirms their potential pathogenic status and risk tendencies in the aquatic environment.

## 5. Limitation of the Study

The few sampling sites in this study may not infer a total representation of the isolates recovered from the province. Subsequent studies should cover additional areas as well as the application of other molecular techniques in the analyses of more virulence signatures among these isolates.

## 6. Conclusions

This study revealed the successful isolation of *V. cholerae*, *V. fluvialis*, *V. vulnificus*, and *V. parahaemolyticus* from SHWE and LCWE, respectively. The molecular analysis revealed that *Vibrio* isolates are an important reservoir of virulence genes critically significant in assessing their pathogenic status. The distribution of virulence genes is valuable for ecological site quality, as well as for epidemiological markers in the control and management of diseases caused by *Vibrio* species. Regular monitoring of HWW and communal wastewater effluent would assist the concerned authorities and policy makers to anticipate, detect, and mitigate any public health threats in advance.

## Figures and Tables

**Table 1:** Sites and the geographical coordinates.

Municipality	Sampling Site	Coordinates
Buffalo City	Tertiary hospital wastewater effluent (THWE)	32°55′37″ S 27°44′42″ E
Amathole District	Secondary hospital wastewater effluent (SHWE)	32°77′53″ S 26°84′64″ E
	Limbede community wastewater (LCWE)	32°77′53″ S 26°84′64″ E

**Table 2:** Oligonucleotide primers for the confirmation of *Vibrio* genus and species.

Target Organism	Gene Target	Oligonucleotide Sequence (5′-3′)	Length (bp)	PCR Cycling Conditions	References
*Vibrio* genus	16S rRNA	FP:CGG TGAAATGCGTAGAGAT RP:TACTAGCGATTCCGAGTTC	663	Firstly, denaturation at 93 °C for 15 min accompanied by 35 cycles of denaturation at 92 °C for 40 s, annealing at 57 °C for 1 min, elongation at 72 °C for 1.5 min and lastly, elongation at 72 °C for 7 min	[31]
*V. cholerae*	*OmpW*	FP:CACCAAGAAGGTGACTTTATTGTG RP:GGTTTGTCGAATTAGCTTCACC	304	Firstly, denaturation at 93 °C for 15 min accompanied by 35 cycles of denaturation: 92 °C for 40 s, annealing: 57 °C for 1 min, elongation 72 °C for 1.5 min, and lastly, elongation at 72 °C for 7 min	[32]
*V. parahaemolyticus*	*flaE*	FP:GCAGCTGATCAAAACGTTGAGT RP:ATTATCGATCGTGCCACTCAC	897	Firstly, denaturation at 94 °C for 5 min, accompanied by 30 cycles of 94 °C for 40 s, 64 °C for 40 s, 72 °C for 90 s, and lastly, elongation at 72 °C for 7 min	[33]
*V. vulnificus*	*Hsp0*	FP: GTCTTAAAGCGGTTGCTGC RP: CGCTTCAAGTGCTGGTAGAAG	410	Firstly, denaturation at 94 °C for 5 min, accompanied by 35 cycles of 94 °C for 30 s, 55 °C for 30 s, 72 °C for 30 s, and lastly, elongation at 72 °C for 10 min	[33]
*V. fluvialis*	*toxR*	FP:GACCAG GGCTTTGAGGTGGAC RP:GGATACGGCACTTGAGTAAGACTC	217	Firstly, denaturation at 94 °C for 5 min, accompanied by 30 cycles of 94 °C for 40 s, 65 °C for 40 s, 72 °C for 1 min, and lastly elongation at 72 °C for 7 min	[34]

**Table 3:** Primer sequences for virulence genes detection among the *Vibrio* species.

Species	Gene	Oligonucleotide Sequence (5′-3′)	Amplicon Size (bp)	Annealing Temp (°C)	References
*V. cholerae*	*tcpA*	F:GAAGAAGTTTRTAAAAGAAGAACA R:GAAAGGACCTTCTTTCACGTTG	451	55	[35]
	*toxR*	F:ATGTTCGGATTAGGACAC R:TACTCACACACTTTGATGGC	779	60	[36]
	*ompU*	F:ACGCTGACGGAATCAACCAAAG R:GCGGAAGTTTGGCTTGAAGTAG	869	62	[37]
	*zot*	F:TCGCTTAACGATGGCGCGTTTT R:AACCCCGTTTCACTTCTACCCA	947	62	[37]
	*ctx*	F:CTCAGACGGGATTTGTTAGGCACG R:TCTATCTCTGTAGCCCCTATTACG	301	55	[38]
	*VPI*	F:GCAATTTAGGGGCGCGACGT R:CCGCTCTTTCTTGATCTGGTAG	680	52	[39]
	*hylA*	F:GAGCCGGCATTCATC TGAAT R:CTCAGCGGGCTAATACGGTTTA	481	60	[40]
*V. vulnificus*	*vcgA*	F:AGCTGCCGATAGCGATCT R:CGCTTAGGATGATCGGTG	278	56	[41]
	*vcgB*	F:CTCAATTGACAATGATCT R:CGCTTAGGATGATCGGTG	278	56	[41]
*V. fluvialis*	*vfh*	F:GCGCGTCAGTGGTGGTGAAG R:TCGGTCGAACCGCTCTCGCTT	800	61	[42]
	*hupO*	F:ATTACGCACAACGAGTCGAAC R:ATTGAGATGGTAAACAGCGCC	600	56	[42]
	*vfpA*	F:TACAACGTCAAGTTAAAGGC R:GTAGGCGCTGTAGCCTTTCA	1790	55	[42]
*V. parahaemolyticus*	*Stn*	F:GGTGCAACATAATAAACAGTCAACAA R:TAGTGGTATGCGTTGCCAGC	375	53	[42]
	*Tdh*	F-GTAAAGGTCTCTGACTTT TGGAC R-TGGAATAGAACCTTCATCTTCACC	269	58	[43]
	*Tlh*	F:AAAGCGGATTATGCAGAAGCACTG R:GCTACTTTCTAGCATTTTCTCTGC	450	58	[44]
	*Trh*	F:TTGGCTTCGATATTTTCAGTATCT R:CATAACAAACATATGCCCATTTCCG	500	58	[43]

## Data Availability

The data and materials used during the current study are available upon request to the corresponding author.
